# Ocean currents and acoustic backscatter data from shipboard ADCP measurements at three North Atlantic seamounts between 2004 and 2015

**DOI:** 10.1016/j.dib.2018.01.014

**Published:** 2018-01-28

**Authors:** Christian Mohn, Anneke Denda, Svenja Christiansen, Manfred Kaufmann, Florian Peine, Barbara Springer, Robert Turnewitsch, Bernd Christiansen

**Affiliations:** a Department of Bioscience, Aarhus University, Frederiksborgvej 399, 4000 Roskilde, Denmark; b Institut für Hydrobiologie und Fischereiwissenschaft, Universität Hamburg, Große Elbstraße 133, 22767 Hamburg, Germany; c GEOMAR Helmholtz Centre for Ocean Research Kiel, Düsternbrooker Weg 20, 24105 Kiel, Germany; d Faculdade das Ciências da Vida, Universidade da Madeira, Portugal; e CIIMAR, Porto, Portugal; f Institut für Biowissenschaften – Meeresbiologie, Universität Rostock, Albert-Einstein-St. 3, 18059 Rostock, Germany; g Scottish Association for Marine Science, Oban PA37 1QA, UK; h CIIMAR-Madeira, Funchal, Portugal

**Keywords:** Seamounts, Northeast Atlantic, Shipboard ADCP, CODAS processing, DIVA gridding

## Abstract

Seamounts are amongst the most common physiographic structures of the deep-ocean landscape, but remoteness and geographic complexity have limited the systematic collection of integrated and multidisciplinary data in the past. Consequently, important aspects of seamount ecology and dynamics remain poorly studied. We present a data collection of ocean currents and raw acoustic backscatter from shipboard Acoustic Doppler Current Profiler (ADCP) measurements during six cruises between 2004 and 2015 in the tropical and subtropical Northeast Atlantic to narrow this gap. Measurements were conducted at seamount locations between the island of Madeira and the Portuguese mainland (Ampère, Seine Seamount), as well as east of the Cape Verde archipelago (Senghor Seamount). The dataset includes two-minute ensemble averaged continuous velocity and backscatter profiles, supplemented by spatially gridded maps for each velocity component, error velocity and local bathymetry. The dataset is freely available from the digital data library PANGAEA at https://doi.pangaea.de/10.1594/PANGAEA.883193.

**Specifications Table**

**Table:** 

Subject area	*Ocean Sciences*
More specific subject area	*Oceanography*
Type of data	*Tabular text files, NetCDF formatted spatial maps*
How data was acquired	*Field surveys, shipboard (system: Teledyne RDI Ocean Surveyor)*
Data format	*Processed, analyzed*
Experimental factors	*N/A*
Experimental features	*Processing of raw single ping data (CODAS toolbox, based on GO-SHIP guidelines for shipboard ADCP data).*
	*Spatial mapping of velocity profiles (DIVA software).*
Data source location	*Field surveys were conducted at three seamounts in the North Atlantic: Ampère Seamount (35° 05′ 0′′ N, 12° 55′ 0′′ W)*
	*Seine Seamount (33° 50′ 0′′ N, 14° 20′ 0′′ W)*
	*Senghor Seamount (17° 10′ 0′′ N, 21° 55′ 0′′ W)*
Data accessibility	*Data is in public repository at*https://doi.pangaea.de/10.1594/PANGAEA.883193

**Value of the data**

• Shipboard ADCP ocean currents and acoustic backscatter data at three different seamounts in the Northeast Atlantic are reported.
• We present fully processed time-averaged continuous velocity profiles and spatially re-gridded velocity fields for each sampling site and period.
• The dataset supports integrated and comparative seamount studies across different physical and biogeographic environments.
• The dataset could be useful for initializing and validating high-resolution hydrodynamic models and species distribution models at seamount relevant spatial scales.

## 1 Data

**Fig. 1:**
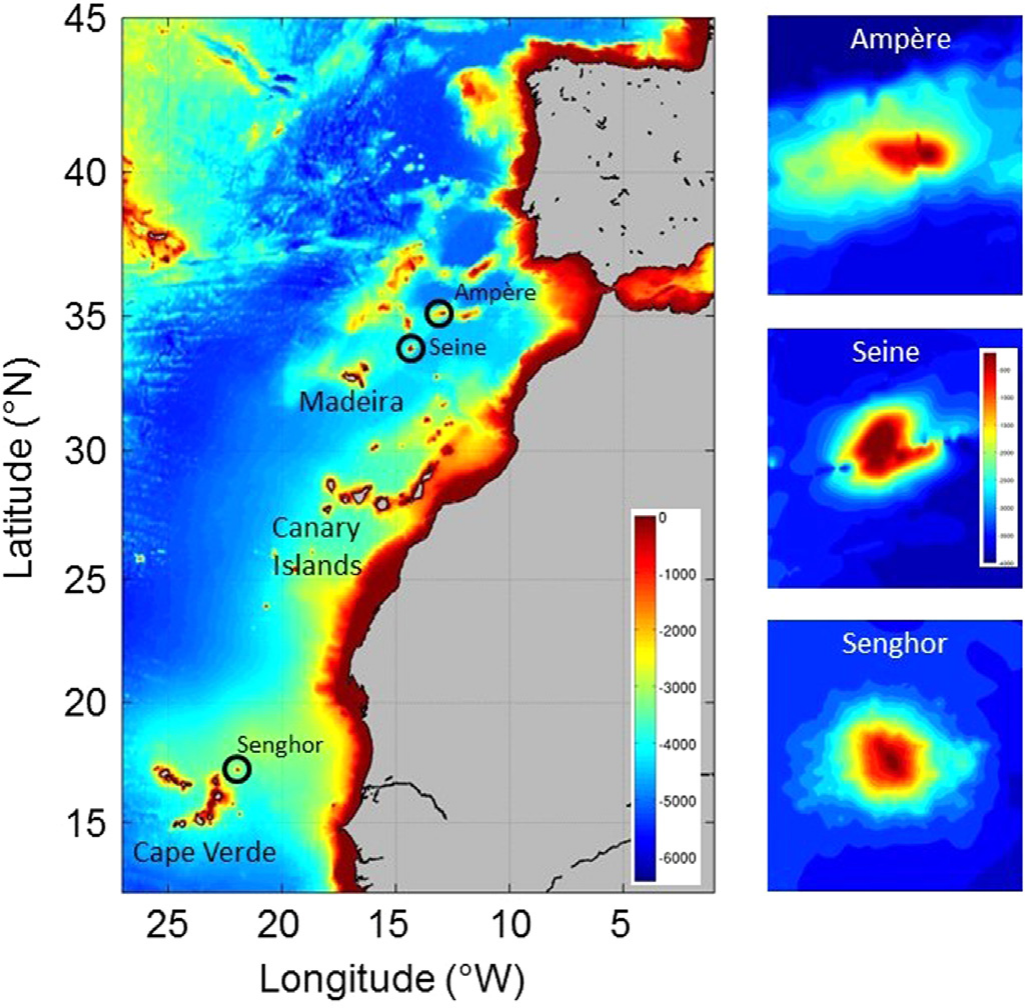
Sampling sites and bathymetry (depths in m) of the Northeast Atlantic based on the Etopo1 1 arc-minute global relief model (left panel). Right panel: Seamount topography (depths in m) based on the Smith and Sandwell (V17.1) 30 arc-seconds global bathymetry (Smith and Sandwell, 1997). Only the depth range 0–4000 m is shown.

**Fig. 2:**
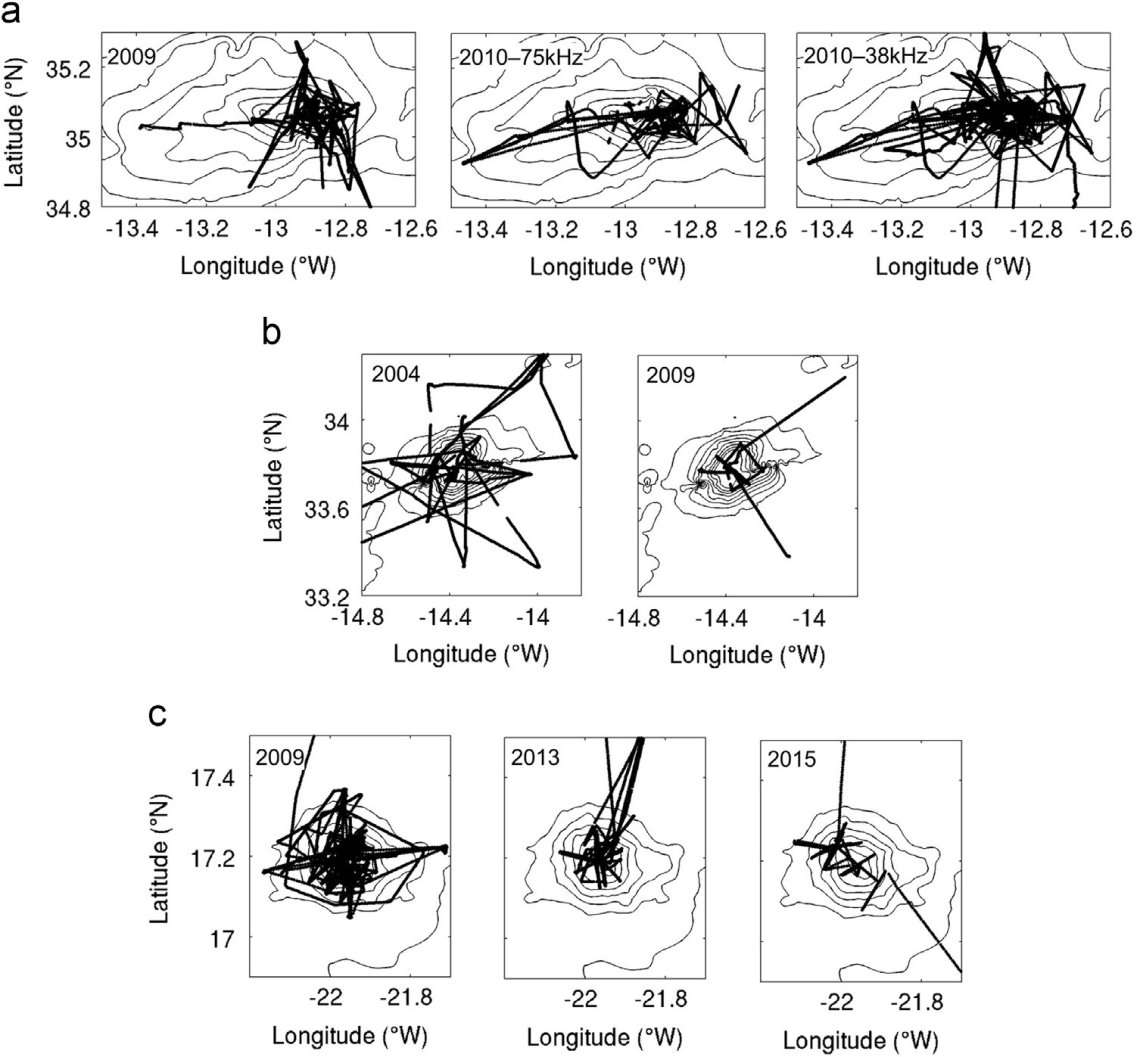
Cruise tracks and locations of 2 min ensemble averaged ADCP profiles for each cruise. (a) Ampère Seamount, (b) Seine Seamount, (c) Senghor Seamount. Cruise details are summarized in Table 1. Black contours indicate seamount topography (depths in m) based on the Smith and Sandwell (V17.1) 30 arc-seconds global bathymetry (Smith and Sandwell, 1997). Only the depth range 500–4000 m is shown (contour interval 500 m).

The dataset presented here was collected during individual field surveys motivated by the demand for integrated and multi-disciplinary data in complex deep-sea environments and the necessity to narrow data gaps in seamount ecosystem research. Tall seamounts ( > 1 km height above the seafloor) are amongst the most prominent features of the global deep-ocean bathymetric landscape. Projected numbers vary between 34.000 [1] and > 100.000 [2]. The large number of tall seamounts in addition to other complex topographic systems including abyssal hills, canyons, ridges and fracture zones interact with currents of different water masses, creating intensified near-bottom flow and mixing that can modify regional deep-sea species diversity and biogeography [3]. Previous and recent studies have highlighted the importance of seamounts as potential biological, biogeochemical and geological hotspots, often externally driven by a wide spectrum of physical processes generated through interactions of oscillatory and steady currents with each unique seamount shape and morphology [4], and references herein]. Such deep-sea hotspots are important for the global carbon and element cycles forming reservoirs of biodiversity and biomass and supporting substantial fisheries. For example, seamounts located within a critical dispersion distance from a shallow-water habitat play an important role as staging posts, supporting meiobenthic steady-state dispersion, assuming they provide similar environmental conditions to the coastal habitats [5]. A mechanistic and quantitative understanding of their influence is, however, still in its infancy. To date, fewer than 200 seamounts have been systematically studied in enough detail to provide integrated assessments of bio-physical connections, ecosystem structure and functioning [6], [7]. Our existing physical knowledge is largely built on theoretical considerations, numerical model studies and results from time-series of single-point current measurements [8]. Direct observations of the spatial structure and variability of flow dynamics at seamounts are rarely available to date, but are fundamental for understanding cycling of organic and sedimentary material at and around seamounts. Here we report on data of currents and raw acoustic backscatter from 8 ADCP surveys, which were conducted during 6 cruises at three Northeast Atlantic seamounts between 2004 and 2015 (Fig. 1, Fig. 2; Table 1). The depth range and vertical bin size varied with ADCP operating frequency and instrument setup, but was typically in the range 20–800 m (8 m bin size, 75 kHz) and 30–950 m (16 m bin size, 38 kHz). The data can be downloaded from the public PANGAEA data repository at https://doi.pangaea.de/10.1594/PANGAEA.883193.

**Table 1:** Cruise details and specifications of instrument configuration and setup in chronological order.

Cruise ID	Seamount	Sampling period	ADCP operating frequency (kHz)	Bin size (m)	Sampled depth range (m)
RRS Discovery D282	Seine	7-July-2004–17-July-2004	75	16	29–845
FS Poseidon P384	Seine	8-May-2009–11-May-2009	75	8	21–765
FS Poseidon P384	Ampère	11-May-2009–20-May-2009	75	8	21–765
FS Meteor M79/3	Senghor	1-Oct-2009–17-Oct-2009	38	16	30–974
FS Meteor M83/2	Ampère	25-Nov-2010–18-Dec-2010	38	16	27–651
		7-Dec-2010–18-Dec-2010	75	8	18–626
FS Poseidon P446	Senghor	6-Feb-2013–14-Feb-2013	75	8	22–750
FS Maria S. Merian MSM49	Senghor	5-Dec-2015–7-Dec-2015	75	5	17–613

## 2 Experimental design, materials and methods

### 2.1 Survey areas

Ampère and Seine Seamount are located between the island of Madeira and mainland Portugal. Ampère Seamount is an elongated seamount centered at 35° 4′ N latitude and 12° 57′ W longitude and extends from 4400 m depth (base diameter 60 × 110 km) to an average summit depth of 120 m with one peak reaching 55 m below sea surface. Seine Seamount is located further to the southwest and closer to the island of Madeira at 33° 50′ N latitude and 14° 20′ W latitude. Seine is a conical seamount and rises sharply from abyssal water depths > 4000 m (base diameter approximately 40 km) to a summit depth of 170 m. Water masses and currents surrounding both seamounts in the upper few hundred meters between the island of Madeira and the Gulf of Cadiz are dominated by the southeastward flowing Azores Currents (AC). The AC is an up to 100 km wide and highly energetic jet centered at 35 °N originating as a branch of the Gulf Stream, crossing the Mid Atlantic Ridge at 35 °N and 45 °W [15] and forms the northern branch of the NE Atlantic subtropical gyre (37–24°N). The NE Atlantic subtropical gyre is characterized by an oligotrophic regime [16], which is reflected by the plankton communities at Ampère and Seine Seamounts. The phytoplankton community comprised mainly nano- and picoplankton (56–95%), which are specialized in low nutrient levels [17]. The faunal composition at the seamounts may be influenced by the dispersal of typical Mediterranean species via the Mediterranean outflow and the formation of meso-scale Mediterranean eddies [18]. Local phenomena driven by tide-topography interaction (e.g. seamount trapped waves and resonantly amplified sub-inertial tidal currents) may also contribute to shape seamount communities at these latitudes.

Senghor Seamount is a nearly axis-symmetric, conically shaped seamount east of the island of Sal, Cape Verde, and is centered at 17° 12′ N and 21° 57′ W. From a small summit plateau at a depth of 100 m it plunges to a base depth of 3300 m (base diameter 35 km). Senghor Seamount is located inside the westward flowing North Equatorial Current (NEC). In the North Atlantic, the NEC extends from 10 ° N to 20 °N [19]. The origin of the NEC is along the NW coast of Africa and it forms the southern branch of the North Atlantic subtropical gyre. Seamount-trapped waters in Taylor caps or tidally-rectified flows were found to be weak and (if existing) limited to depths > 250 m, but energetic and freely propagating internal waves were described along the upper northern and southern slopes of the seamount [9]. The seamount plankton community is therefore expected to be strongly influenced by large-scale flow features, such as seasonal filaments of the Mauritanian upwelling [20] and the Cape Verde frontal zone (CVFZ, [21]). The waters of the NEC south of the CVFZ, enclosing Senghor Seamount, are considered as nutrient-rich [22] with a strong influence of the Mauritanian upwelling [23], [24]. Relatively high nutrient concentrations were measured over Senghor Seamount, indicating diatoms and dinoflagellates to be more abundant than nano- and picoplankton, which dominated the phytoplankton community at Ampère. Consequently, in terms of biomass the ratio of mesozooplankton to microzooplankton was higher at Senghor than at Ampère [10], [25].

### 2.2 ADCP data collection and processing

ADCP data were collected with 38 and 75 kHz Teledyne RDI Ocean Surveyor (OS) systems in the upper 900 m of the water column. Single ping velocity profiles were recorded together with corresponding records of ship position, ship heading and time. RDI's VmDAS software package was used to set instrument configuration, data communication, and data acquisition (see Table 1 for specifications of the ADCP setup during different cruises). A number of environmental and technical factors including sound absorption, lack or excess of scatterers, misaligned transducer orientation relative to the ship and ship motion can adversely affect the quality of ADCP measurements [11]. ADCP data processing is therefore necessary to transfer single ping ADCP raw data into accurate and realistic estimates of ocean currents. We used the Common Oceanographic Data Access System [12], available online at http://currents.soest.hawaii.edu/docs/adcp_doc/index.html) for data processing following the GO-SHIP guidelines for shipboard ADCP measurements [11]. As a first processing step, single ping raw ADCP velocity profiles were time-averaged into 2 min ensembles. An averaging period of 2 min was considered a reasonable trade-off between the need to reduce random noise and maintaining high along-track resolution at the same time. In a second step, ensemble velocity profiles were quality controlled. We discarded depth bins with percent good values ≤ 20% of the return signal. Percent good is measure of data quality and defines the ratio of good pings per total pings for each ensemble profile. Low beam correlation and large error velocity are possible sources for low percent good values. A water track calibration was performed for each dataset to obtain accurate estimates of flow magnitude and direction by assessing transducer orientation relative to the ship's keel and velocity amplitude scale factors. Finally, absolute current velocities were calculated by removing the best estimate of ship velocities for each ensemble from the corresponding relative ADCP velocities. Major error sources in determining absolute shipboard ADCP velocities result from remaining bias in estimating the transducer misalignment (phase) and the velocity amplitude scale factor. Phase errors of < 0.2° result in ADCP velocity errors of 2 cm s^−1^ and amplitude scale factor errors of 0.5% generate velocity errors 2–3 cm s^−1^, respectively. The instrument's measurement accuracy is 0.5 cm s^−1^ according to the manufacturer's data. ADCP data from all surveys have been checked and corrected for possible phase and amplitude errors and remaining errors after processing were within the above limits.

### 2.3 Re-gridding of the spatially non-uniform data

Adjustments of the planned sampling programme due to weather conditions and scientific needs often lead to an asynoptic and spatially scattered cruise track and, in consequence, to an heterogeneous data distribution. In addition, the along ship-track resolution of the ADCP data is usually much higher than the resolution between tracks. To compensate for part of this time-space bias in the data, ADCP velocity components were spatially re-gridded at each depth bin in an optimal way using the Matlab interface of the DIVA (Data Interpolating Variational Analysis) optimal interpolation and error analysis toolbox [13]. The target grid size was set to 30 arc seconds (1/120° or approximately 0.5 nautical miles) in latitude and longitude and the spatial correlation length scale (L) was set to L = 0.2° representing a characteristic seamount length scale in all areas. The signal to noise ratio λ was set to a constant value of order unity (λ = 1) to minimize artificial values in the gridded velocities due to the spatial resolution mismatch between along- and cross-track ADCP data. The 30 arc second Smith and Sandwell global topography data set V17.1 [14] was applied to mask areas, where the bottom topography intersects each respective depth level. To provide confidence estimates for the DIVA analysis, the relative error was calculated along with the gridded velocity fields for each survey based on a data covariance function [13].

### 2.4 Data structure

**Fig. 3:**
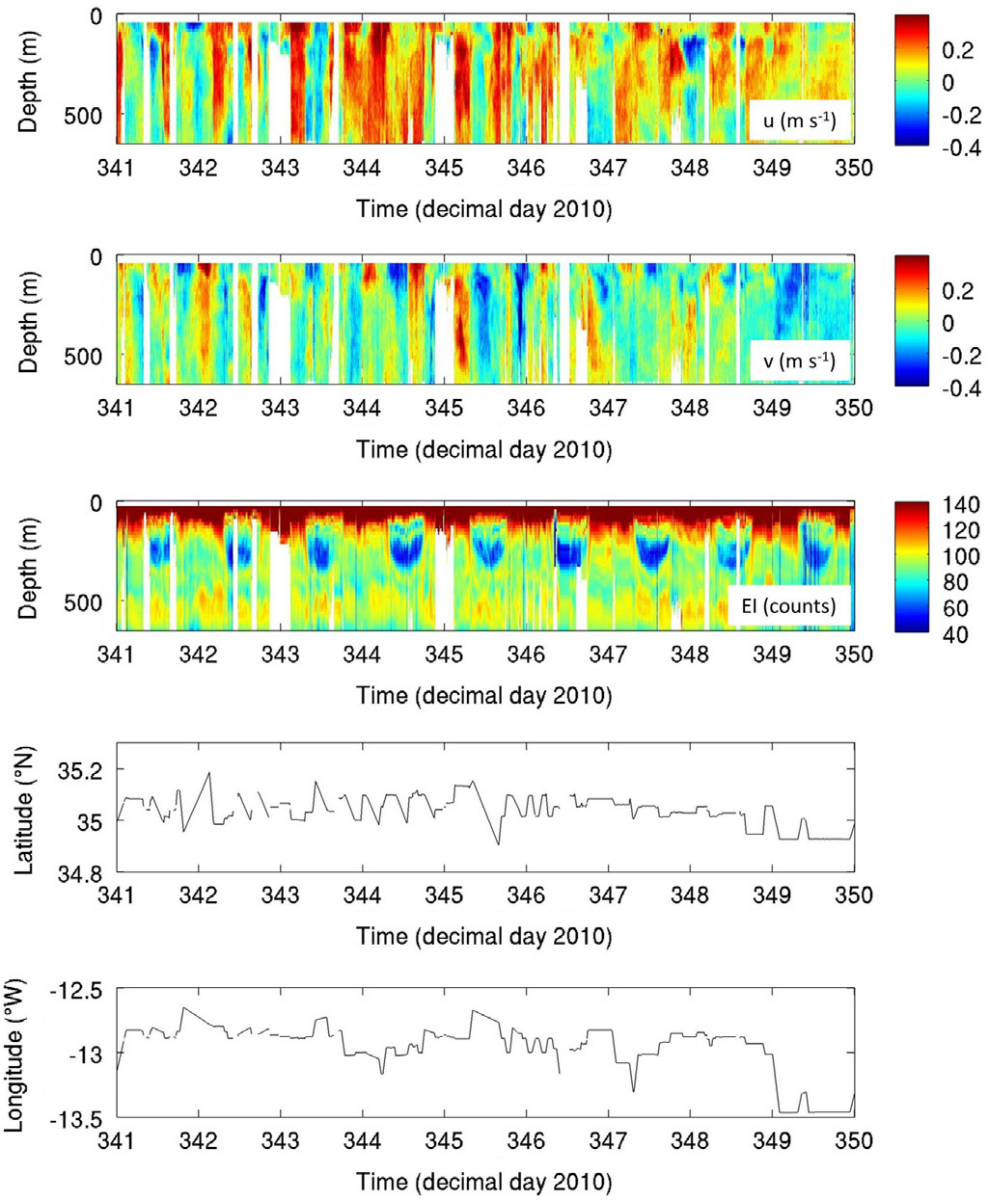
Partial time series of velocity components u, v (m s^−1^), echo intensity (raw counts) and cruise track at Ampère Seamount from the FS Meteor M83/2 cruise (Nov/Dec 2010, 38 kHz ADCP). Depth bins with percent good values < 20% and/or missing data are not included.

**Fig. 4:**
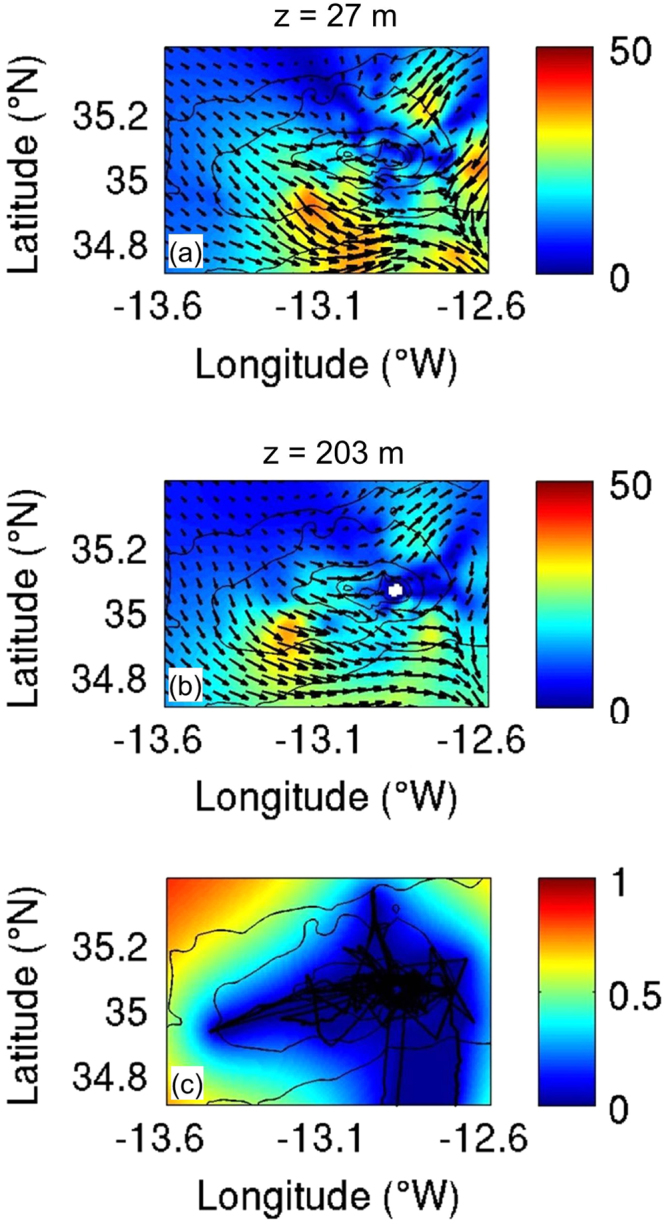
Composite plots of DIVA gridded ADCP current speed (coloured contours) and current direction (vectors) at the depth levels 21 m (a) and 197 m (b). The relative error field is shown in (c). Plots are based on results from the FS Meteor M83/2 cruise (Nov/Dec 2010, 38 kHz ADCP) at Ampère Seamount. Current speeds were calculated from the individual velocity components according to $U=\sqrt{u^{2}+v^{2}}$ (U = current speed in cm s^−1^, u and v are the East-West and North-South velocity components in cm s^−1^). The error fields include the location of the original ADCP profiles. Depth contours 500, 1000, 2000, 3000 m of seamount bathymetry are superimposed.

Each dataset includes two components. The first component includes 2-minute ensemble averaged continuous profiles of both horizontal velocity components (u and v in m s^-1^), along with corresponding profiles of relative acoustic backscatter (echo intensity in counts) and the data quality parameter percent good for each depth bin and geographic position (see a partial time series example in Fig. 3). Profile data are presented in a tabular, column-oriented text file with variables in each column and time records in each row. The variables are date (yy-mm-dd), time (HH:MM:SS), decimal day, longitude (°), latitude (°), depth (m), u-velocity (m/s), v-velocity (m/s), echo amplitude (raw counts) and percent good. The second component reports the DIVA spatially re-gridded velocity and relative error fields together with the seamount topography used for data masking in NetCDF format. NetCDF is a self-describing and platform independent data format. NetCDF formatted files can be accessed by graphical NetCDF file browsers (ncBrowse, ncview) and NetCDF interfaces provided in software environments for mathematical and statistical computing such as Matlab and the R project. Fig. 4 presents composites of gridded velocity fields at two different depth levels from 38 kHz ADCP data collected during the FS Meteor 83/2 cruise (November/December 2010) at Ampère Seamount.
